# Upper Limb Compartment Syndrome—An Extremely Rare Life-Threatening Complication of Cutaneous Anthrax

**DOI:** 10.3390/microorganisms12061240

**Published:** 2024-06-20

**Authors:** Mihaela Pertea, Stefana Luca, Dan Cristian Moraru, Bogdan Veliceasa, Alexandru Filip, Oxana Madalina Grosu, Vladimir Poroch, Andrian Panuta, Catalina Mihaela Luca, Andrei Nicolae Avadanei, Sorinel Lunca

**Affiliations:** 1 Department Plastic Surgery and Reconstructive, Faculty of Medicine, “Grigore T. Popa” University of Medicine and Pharmacy, 700115 Iasi, Romania; mihaela.pertea@umfiasi.ro (M.P.); stefana.luca@d.umfiasi.ro (S.L.); cristian-dan.moraru@umfiasi.ro (D.C.M.); oxana-madalina.grosu@umfiasi.ro (O.M.G.); 2 Department of Plastic Surgery and Reconstructive Microsurgery, “Sf. Spiridon” Emergency County Hospital, 700111 Iasi, Romania; andrian.s.panuta@umfiasi.ro; 3 Department of Orthopaedics and Traumatology, Faculty of Medicine, “Grigore T. Popa” University of Medicine and Pharmacy, 700115 Iasi, Romania; alexandru-filip@umfiasi.ro; 4 Department of Orthopaedics and Traumatology, “Sf. Spiridon” Emergency County Hospital, 700111 Iasi, Romania; 5 Department of Palliative Care, “Grigore T. Popa” University of Medicine and Pharmacy, 700115 Iasi, Romania; vladimir.poroch@umfiasi.ro; 6 Palliative Oncological Clinic, Regional Institute of Oncology, 700483 Iasi, Romania; 7 Department of Surgery I, Faculty of Medicine, “Grigore T. Popa” University of Medicine and Pharmacy, 700115 Iasi, Romania; sorinel.lunca@umfiasi.ro; 8 Department of Infectious Diseases, “Grigore T. Popa” University of Medicine and Pharmacy, 16 Universitatii Street, 700115 Iasi, Romania; catalina.luca@umfiasi.ro; 9 Clinic of Infectious Diseases, “Sf. Parascheva” Clinical Hospital of Infectious Diseases, 700116 Iasi, Romania; 10 Department of Vascular Surgery, Faculty of Medicine, “Grigore T. Popa” University of Medicine and Pharmacy, 700115 Iasi, Romania; andrei.avadanei@umfiasi.ro; 11 Department of Vascular Surgery, “Sf. Spiridon” Emergency County Hospital, 700111 Iasi, Romania; 12 Second Oncological Clinic, Regional Institute of Oncology, 700483 Iasi, Romania

**Keywords:** cutaneous anthrax, compartment syndrome, surgery, antibiotics

## Abstract

(1) Background: Cutaneous anthrax is a disease caused by a Gram-positive bacillus, spore-forming *Bacillus anthracis* (BA). Cutaneous anthrax accounts for 95% of all anthrax cases, with mortality between 10–40% in untreated forms. The most feared complication, which can be life-threatening and is rarely encountered and described in the literature, is compartment syndrome. (2) Methods: We report a series of six cases of cutaneous anthrax from the same endemic area. In two of the cases, the disease was complicated by compartment syndrome. The systematic review was conducted according to systematic review guidelines, and the PubMed, Google Scholar, and Web of Science databases were searched for publications from 1 January 2008 to 31 December 2023. The keywords used were: “cutaneous anthrax” and “compartment syndrome by cutaneous anthrax”. (3) Results: For compartment syndrome, emergency surgical intervention for decompression was required, along with another three surgeries, with hospitalization between 21 and 23 days. In the systematic review, among the 37 articles, 29 did not contain cases focusing on compartment syndrome of the thoracic limb in cutaneous anthrax. The results were included in a Preferred Reporting Items for Systematic Reviews and Meta-Analysis (PRISMA) flow diagram. (4) Conclusions: Early recognition of the characteristic cutaneous lesions and compartment syndrome with early initiation of antibiotics and urgent surgical treatment is the lifesaving solution.

## 1. Introduction

Anthrax is known to be a highly aggressive infection (zoonosis) with high virulence and contagiosity, capable of causing death. The first description of this disease was written by the Roman poet Virgil [1]. The disease is caused by a Gram-positive bacillus, spore-forming *Bacillus anthracis* (BA). It can survive in soil and animal products for years, with extremely high contagiosity. Human-to-human transmission has not been confirmed. Transmission of this infection to humans occurs from infected animals through direct exposure or contact with their products: wool, skin, or meat. Among the animals most exposed to anthrax infection are cattle, sheep, goats, horses, and pigs. Possible infection through insect stings has also been reported [2]. It has been proven that amphibians and reptiles are resistant to *Bacillus anthracis* infection, having cold blood [3]. With warming, experimentally, these creatures’ resistance decreases, making them susceptible to anthrax infection. There are several professional categories with a high risk of BA infection: farmers, livestock breeders, butchers, and veterinarians. Depending on the route of contamination, there are three main forms of anthrax: cutaneous, gastrointestinal, and respiratory (pulmonary) [4,5]. Since 2000, with the description of the first case of death caused by an injection of heroin contaminated with anthrax through skin-popping, in a drug user, the term “injectional anthrax” has been used [6,7]. Cutaneous anthrax accounts for 95% of all anthrax cases, with mortality of 10–40% in untreated forms and decreasing to less than 1% in correctly treated cases [8]. In most cases, the cutaneous form self-limits under treatment, evolving without complications. Cutaneous anthrax lesions are typically located on exposed anatomical regions: thoracic limbs, neck, or cephalic extremities. The characteristics of anthrax cutaneous lesions can be described as pathognomonic, initially described as a painless, pruritic pustule appearing after contact with an infected animal or its products [9]. The evolution of the lesion is rapid; on erythematous skin; around the papule, a vesicular border develops, the central area of the papule becomes depressed, and in a short time is covered by a black hemorrhagic crust (eschar). From this evolutionary moment, the lesion can become superinfected or, conversely, evolve favorably, drying out and spontaneously detaching, leaving behind a scar [10]. The most life-threatening complications of the disease are meningoencephalitis and septic shock, with a mortality rate of 100% [11]. In the case of cutaneous anthrax, the most feared complication, which can be life-threatening and is rarely encountered and described in the literature, is compartment syndrome [10]. Compartment syndrome is a serious condition characterized by increased intracompartmental pressure with damage to the vascularization of the affected limb segment, clinically characterized by pain, pallor, paresthesia, pulselessness, and paralysis. This extremely rare complication must be known to clinicians in endemic areas (Sub-Saharan Africa, Central and South America, Middle East, India) as well as outside these areas [12]. Although surgical intervention in the case of cutaneous anthrax lesions has a related contraindication, as it can cause distant dissemination and sepsis, in cases with compartment syndrome, it is an absolute indication, constituting a major surgical emergency [13]. It should not be forgotten that, besides “natural” contamination, BA can be one of the most important agents used in bioterrorism in the event of a biological war, as it was used in the United States in 2001 (Klietmann and Ruoff) [14]. In this context, even “natural” cases of anthrax must be reported and recorded at the national level. The current study presents a series of six cases of cutaneous anthrax, two confirmed and four suspected (according to the case definition), in two of which compartment syndrome was present. All the cases in the series come from the same area, with contamination occurring in the same epidemiological context. A systematic review was conducted by studying all reports of cutaneous anthrax complicated with compartment syndrome in the literature starting from 2008.

## 2. Materials and Methods

### 2.1. Case Series

We studied a series of 6 cases consulted in the Plastic Surgery and Reconstructive Microsurgery Department of “Sf Spiridon” Emergency County Hospital, Iasi, Romania, between July and August 2023. Two of the six cases were admitted and treated in the same clinic, and four of them were referred for specialized treatment to the Infectious Diseases Hospital, Iasi. In all the cases, the patients were informed about the disease and the necessary medical and surgical treatment to be administered and signed informed consent. For the publication of the current study, the approval of the Ethics Committee of “Sf Spiridon” Hospital, Iasi, Romania, no. 11/date 26 February 2024, was obtained. The inclusion criteria of the cases in the study complied with the case definition for cutaneous anthrax. In the two cases where compartment syndrome was diagnosed, urgent surgical decompression was indicated. The surgical procedures were performed under general anesthesia with orotracheal intubation (OTI).

### 2.2. Data Collected and Systematic Review Method

The data were collected following the Preferred Reporting Items for Systematic Reviews and Meta-Analyses guidelines. The articles included in the review followed the following inclusion criteria: English language, publication in any journals, any reporting type article, original, review, or case presentation. Further exclusion criteria were books, editorial comments, notes, etc. Furthermore, we arbitrarily decided to start our research from 1 January 2008. This systematic review was conducted according to the Systematic Reviews guidelines, and the PubMed, Google Scholar, and Web of Science databases were searched for publications from 1 January 2008 to 31 December 2023. The keywords used were: “cutaneous anthrax”, “compartment syndrome by cutaneous anthrax”, and both (Table 1).

Articles were excluded by the language in which they were reported (any other than English), title (another type of anthrax with a different localization than the thoracic limbs), abstract, or full text for the processes connected to the subject of the manuscript and for irrelevance to the topic in question (compartment syndrome in cutaneous anthrax at the level of the thoracic limb), and pediatric cases. For the inclusion of the articles in the systematic review, the following were recorded: the studied group (number of cases, sex, age), mode of contamination, incubation period, location and number of lesions, and signs and symptoms including complications—compartment syndrome, paraclinical explorations, results of the examination of the secretions collected from the lesions, how to diagnose anthrax, antibiotic or surgical treatment, and evolution.

## 3. Results

### 3.1. Case Report 1

**Figure 1:**
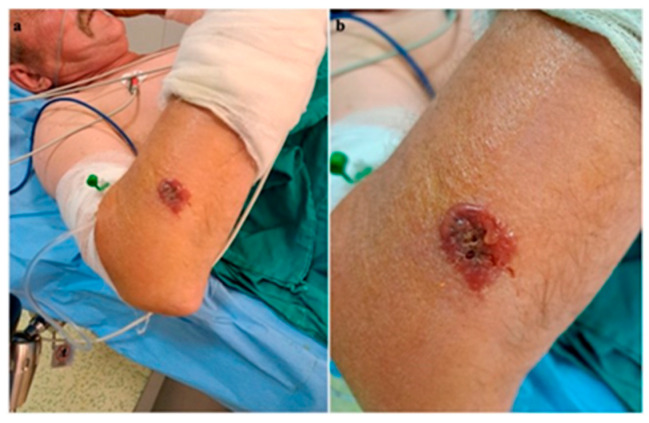
(**a**) Right forearm cutaneous anthrax lesion, (**b**) lesion detail.

A 56-year-old male patient presented to the emergency department with a fever of 40 °C, fainting spells, and cutaneous lesions on both forearms: a single pruritic lesion on the right forearm, at the middle third of the dorsal forearm, resembling a delimited papule surrounded by a ring of vesicles containing clear liquid. The center of the papule was slightly depressed, centered by an area of necrosis (Figure 1a,b).

**Figure 2:**
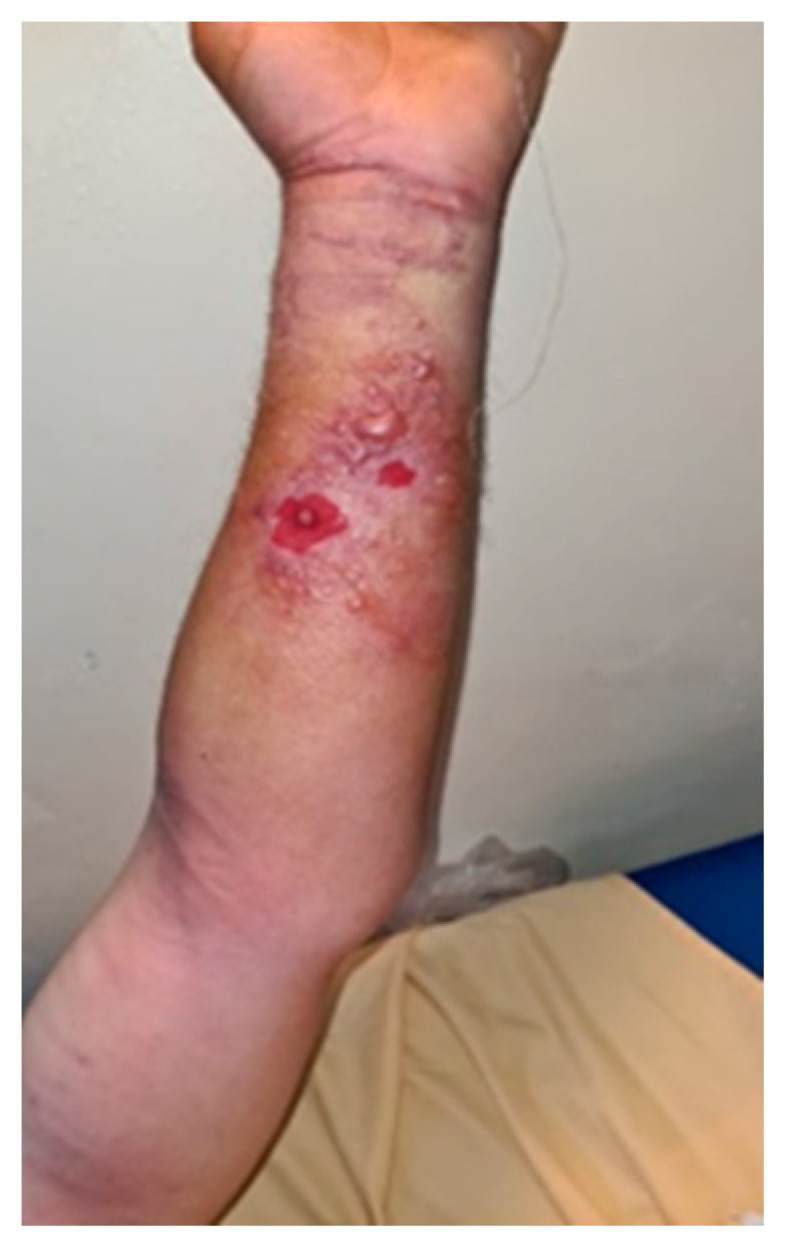
Initial left forearm injury—day 1.

On the left forearm at the level of the wrist and the distal third of the forearm, on the volar face, there was itchy, erythematous skin with lesions traumatized by the patient, presenting an ovoidal denuded area, centered by hardening of the same aspect (Figure 2).

**Figure 3:**
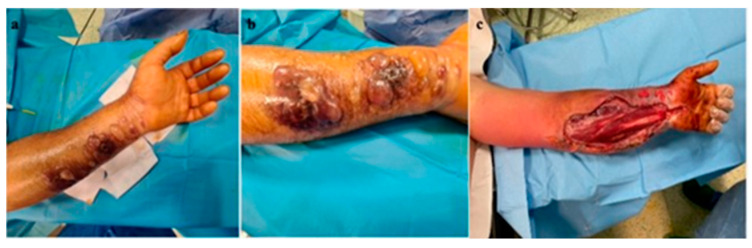
(**a**) Cutaneous anthrax case 1 with compartment syndrome, (**b**) cutaneous anthrax case 1 with compartment syndrome—detail, (**c**) decompression incisions for surgical treatment of compartment syndrome; significant erythema and edema at the level of the left thoracic limb.

The patient, a livestock farmer, reported contact 7 days previously with cattle. The general condition of the patient, medical history, and characteristics of the lesions led to suspicion of cutaneous anthrax, for which he was referred to the Infectious Diseases Hospital, where antibiotic treatment with penicillin G (6 million units/day) and ciprofloxacin (400 mg/day) was initiated. The patient was reviewed by the plastic surgery service 10 hours after the initial consultation, when the extension of cutaneous lesions on the left forearm was observed, with the appearance of blisters containing clear liquid, with color changes of the volar forearm skin, tense skin, sensitivity changes (hypoesthesia) in the territory of the median and ulnar nerves, cold skin of the hand, absent capillary refill in the fingers, and absent pulse at the radial and ulnar arteries at the wrist level. The erythema and significant edema extending to the left axilla were noted. The diagnosis of compartment syndrome was established, and surgical intervention was indicated despite the presence of risks (bacterial dissemination, septic shock). Biological examination showed a fever of 39.6 °C, fainting, severe headache, incoherent speech, white blood cells (WBC) = 19.73 × 103/µL, and CRP = 3.95 mg/dL. Surgical intervention under general anesthesia with endotracheal intubation was performed, collecting the secretions from the blister content, performing decompression incisions at the level of the forearm with the opening of the anterior annular ligament of the carpus (carpal tunnel) with their extension to the distal third of the arm, and necrosectomy (Figure 3a,b). There were no organized or diffused purulent collections at the level of the forearm.

Skin, muscle, and vascular biopsies were collected for histopathological examination. The initial antibiotic treatment continued under the monitoring of the infectious disease specialist. Leukocytosis remained elevated but started to decreased after the 4th day of general treatment (Table 2).

**Figure 4:**
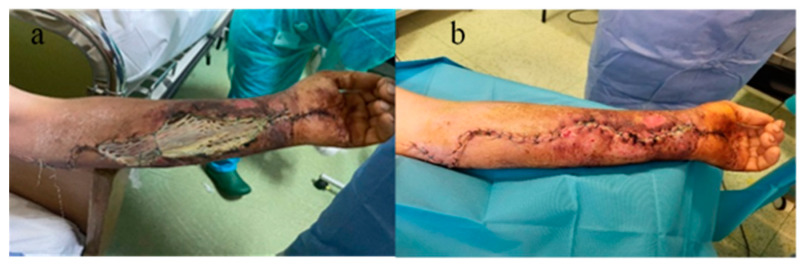
(**a**) Failure of split skin graft, (**b**) direct closure of the decompression incisions after edema remission.

Local cleansing and daily dressings were performed. Significant exudation was observed at the sites of decompression incisions. On the 7th day after surgical intervention, the remaining skin defect was covered with a free skin graft harvested from the ipsilateral thigh, but due to the moist environment, the split skin graft failed. General antibiotic treatment was continued, with the patient receiving analgesic and steroid anti-inflammatory treatment (dexamethasone). The patient’s general condition improved, and the erythema and edema of the left thoracic limb reduced, so that on the 10th day after admission, under intravenous sedation, secondary suturing (direct closure) of the decompression incisions was performed (Figure 4a,b).

**Figure 5:**
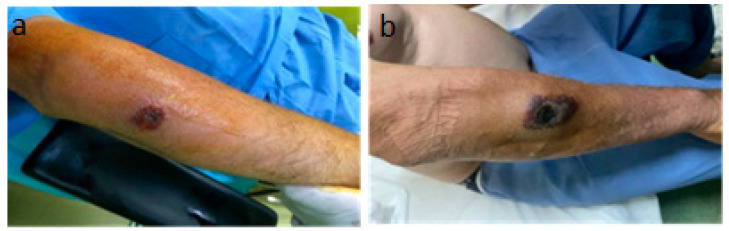
The progressive but self-limited evolution of the injury in the right forearm. (**a**) The 5th day of evolution, (**b**) the 20th day of evolution.

As for the cutaneous lesion on the dorsal aspect of the right forearm, it changed in size and appearance but without causing complications, limiting itself over time (Figure 5a,b).

**Figure 6:**
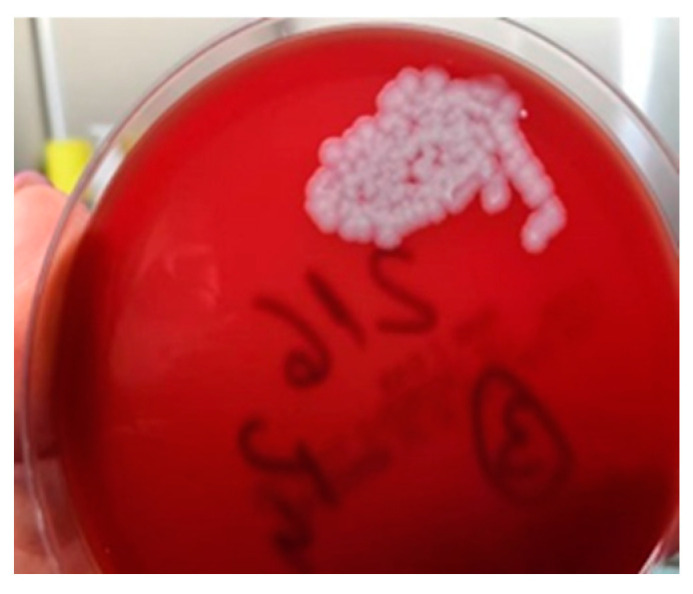
*Bacillus anthracis* culture.

The method used to determine BA was the Micronaut System (MALDI-TOF and microdilution in plates). Biopsy specimens collected at admission showed Gram-positive bacilli suggestive of the genus *Bacillus* spp., but the cultures remained negative. At subsequent surgical interventions, sampling and seeding were performed directly in the operating room; microscopic examination again showed Gram-positive bacilli, and the cultures grew non-hemolytic, rough colonies suggestive of *Bacillus* spp. (Figure 6). The strains were sensitive to penicillin. A differential diagnosis was made of *Bacillus cereus*. 

**Figure 7:**
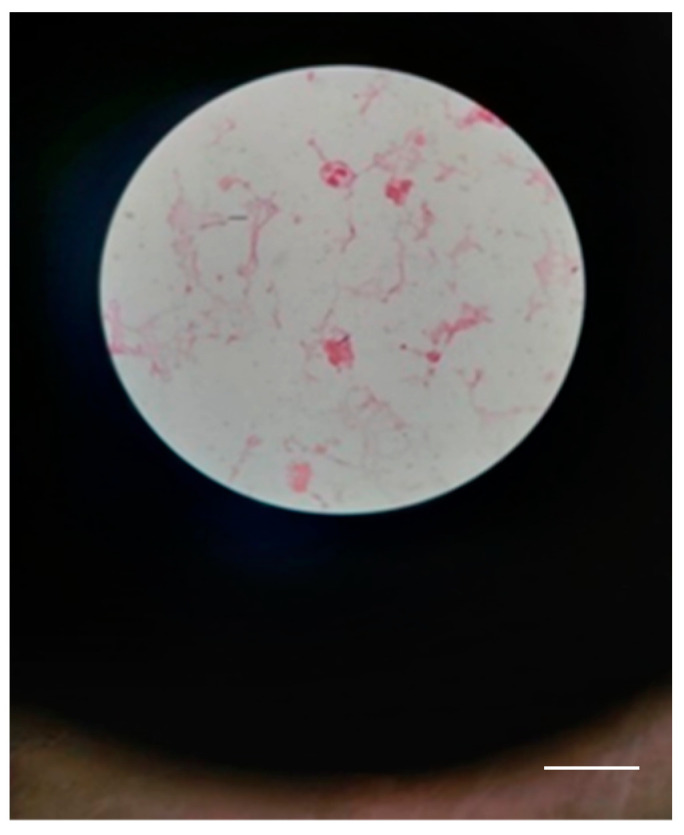
Few Gram-positive bacilli with truncated ends in a case 1 (scale bar: 10 μm).

The definitive identification of the species could not be performed because the database of the MALDI-TOF system is restricted in identifying bioterrorism agents in class II biosafety laboratories. The samples were sent to the Institute of Diagnostic and Animal Health in Bucharest, Romania, which confirmed the presence of *Bacillus Anthracis* (Figure 7).

**Figure 8:**
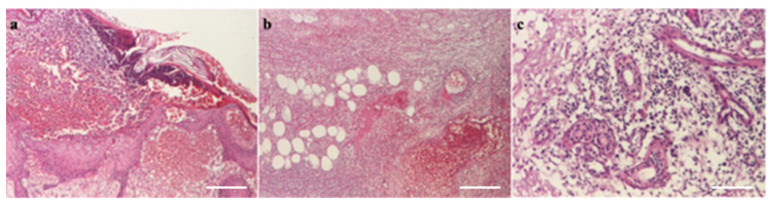
(**a**) Pustule (HEx40) scale bar: 250 μm, (**b**) necrosis with hypodermic thrombosis (HEx40) scale bar: 250 μm, (**c**) periaxial inflammation (HEx100) scale bar: 100 μm.

A histopathological examination revealed almost total ulceration of the epidermis, extensive ischemic necrosis, areas of recent hemorrhage, abundant polymorphic inflammatory infiltrates with abscess foci, and some recent vascular thromboses, consistent with an acute inflammatory process of the necrotizing cellulitis type (Figure 8). Microbial colonies of positive cocci and scattered bacilli with truncated ends were observed in the Gram stain. PAS, Grocott, and Giemsa stains did not identify specific microorganisms.

The patient was discharged after 21 days of hospitalization in good general condition, without any functional impairment of the operated thoracic limb, with recommendations for further follow-up and monitoring by the family doctor.

### 3.2. Case Report 2

**Figure 9:**
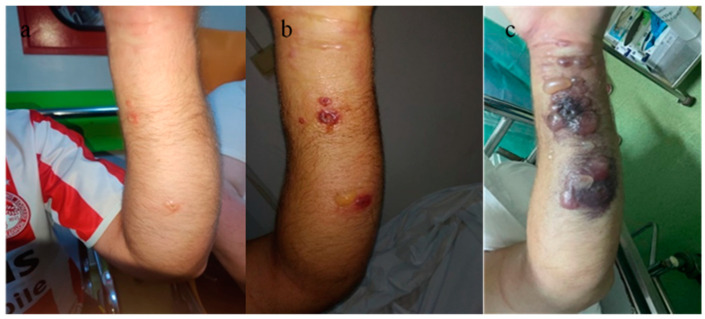
Cutaneous anthrax. Characteristic skin lesions evolving within a 3-day interval. (**a**) Aspect on admission, (**b**) aspect on the 2nd day after admission, (**c**) 3rd day after admission, with the onset of compartment syndrome and the appearance of characteristic lesions for cutaneous anthrax.

A 46-year-old male patient was admitted to the same plastic surgery department, five days after the admission of the previous case. On admission, the patient had a fever, chills, and multiple cutaneous lesions on the volar aspect of the left forearm. Initially, the lesions were small, erythematous, pruritic papules, with a diameter of 0.5 cm, centered by a depressed area. Moderate edema was observed at the forearm level. From the patient’s history, the same epidemiological context was observed, the patient having the same occupation as the first case and coming from the same geographical region. The diagnosis of anthrax was suspected, and antibiotic treatment similar to the first patient of Penicillin (6 million units/day) and ciprofloxacin (400 mg/day) was initiated. This treatment lasted for 7 days, after which, at the infectious disease specialist’s recommendation, the penicillin was replaced with Amoxicillin plus 1.2 g × 3 for another 14 days. The patient was monitored in the plastic surgery department. The evolution of the cutaneous lesions to the stage of large blisters, with extensive necrotic areas, led to the clinical diagnosis of compartment syndrome on the 3rd day after admission (Figure 9).

Surgical intervention under general anesthesia and endotracheal intubation was decided in this case as well. Secretions were collected for bacteriological examination. Decompression incisions were made at the forearm level, with an opening of the anterior ligament of the wrist (carpal tunnel) and proximally to the elbow fold. Skin, vascular, and fascial biopsies were collected for histopathological examination. On admission, leukocytosis was 13.35 and CRP = 3.75, with fluctuating evolution. 

**Figure 10:**
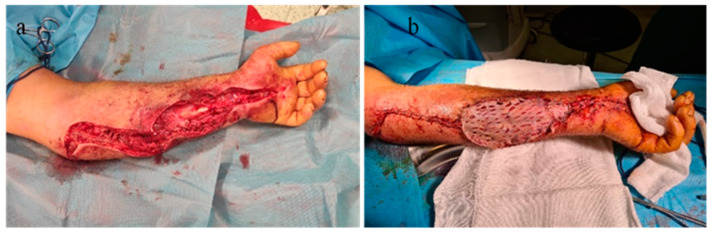
(**a**). Decompression incisions in compartment syndrome. (**b**) Closing and covering the postexcisional soft tissue defect by plasty with split skin graft after edema remission.

Dressings with antiseptic solutions on the postoperative wound as well as the antibiotic treatment instituted from the patient’s admission to the hospital were continued throughout the 22-day hospitalization period. On the 8th day after surgical intervention, it was decided to close the soft tissue defect (under general anesthesia with endotracheal intubation) by direct suture with separate stitches at the carpal canal and in the proximal portion of the forearm, and, in the middle third, split-thickness skin grafting was performed with skin harvested from the contralateral thigh of the affected thoracic limb (Figure 10).

**Figure 11:**
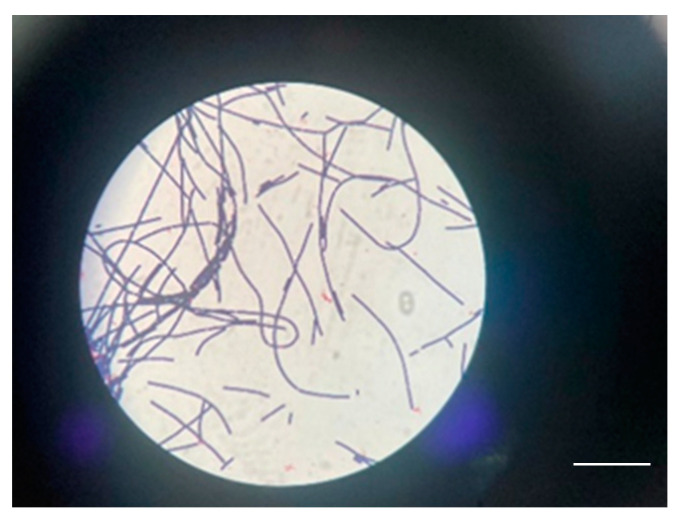
Gram-positive bacilli with truncated ends in a case 2 scale bar: 100 μm.

A bacteriological examination of the secretion collected from the blisters, using mass spectrometry (MALDI-TOF), showed the possible presence of BA. On the Gram-stained smears, a few leukocytes were observed, along with Gram-positive bacilli with truncated ends (Figure 11).

**Figure 12:**
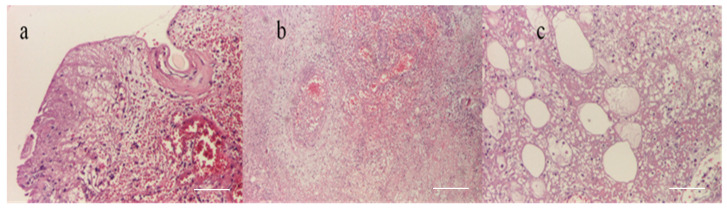
(**a**) Epithelium with coagulation necrosis (HEx100) scale bar: 100 μm, (**b**) dermis with coagulation necrosis (HEx40) scale bar: 250 μm, and (**c**) hypodermis with coagulation necrosis (HEx100) scale bar: 100 μm.

A histopathological examination of the entry site revealed areas of ischemic necrosis with a fibrino-leukocyte exudate on the surface, rich polymorphous inflammatory infiltrates, vessels of fibrinoid necrosis and neutrophils (vasculitis), some recent thrombosis, areas of liponecrosis and lipogranulomatosis, and recent and marked congestive hemorrhage (Figure 12). PAS and Giemsa staining highlighted the presence of spores and articulated bacilli arranged in short chains.

The patient was discharged after 22 days of hospitalization without any signs of functional impairment at the level of the thoracic limb, with recommendations for physiotherapy and further follow-up by the family doctor. Both patients received recommendations from the infectious disease specialist to continue antibiotic treatment for 10 days with penicillin (6 million units/day) and ciprofloxacin (400 mg/day) at the Infectious Diseases Department.

### 3.3. Case Reports 3–6

**Figure 13:**
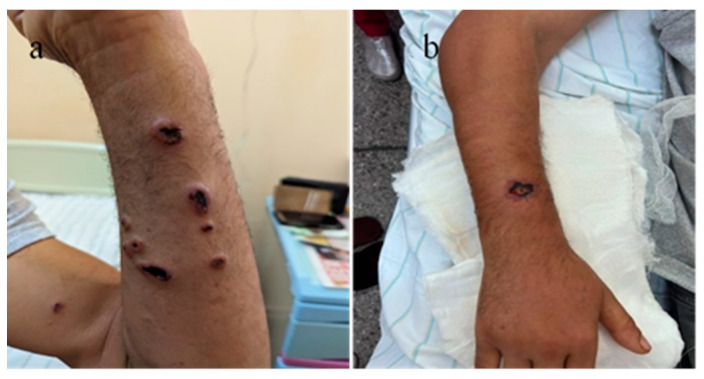
(**a**). Multiple self-limited cutaneous anthrax lesions on the left forearm, (**b**) single cutaneous anthrax lesion on the dorsal aspect of the right forearm after an initial excision.

During the hospitalization of the first two cases, four additional cases of male patients from the same geographical region, with the same occupation and under the same epidemiological context, presented at the emergency unit. All four patients exhibited characteristic multiple cutaneous anthrax lesions on their forearms, following minor traumas due to contact with products from dead animals under unknown conditions (Table 3, Figure 13). In one of these cases, incomplete patient statements regarding the epidemiological context led to an initial misdiagnosis as a simple puncture wound, resulting in surgical excision. 

However, the subsequent evolution of the lesion, combined with the patient’s statements about the mode and location of the injury, directed the diagnosis towards cutaneous anthrax (according to the definition of a suspicious case), and the patient was referred to the Infectious Diseases Hospital for specialized treatment (Table 3) (Figure 13).

In all the cases, specific antibiotic treatment was initiated, with ciprofloxacin (400 mg/day) and cefort (4 g/day for 10 days), resulting in favorable evolution with self-limitation of cutaneous lesions without local or general complications. Patients were discharged after an average hospitalization period of 10 days in an improved condition.

### 3.4. Results—Systematic Review

**Figure 14:**
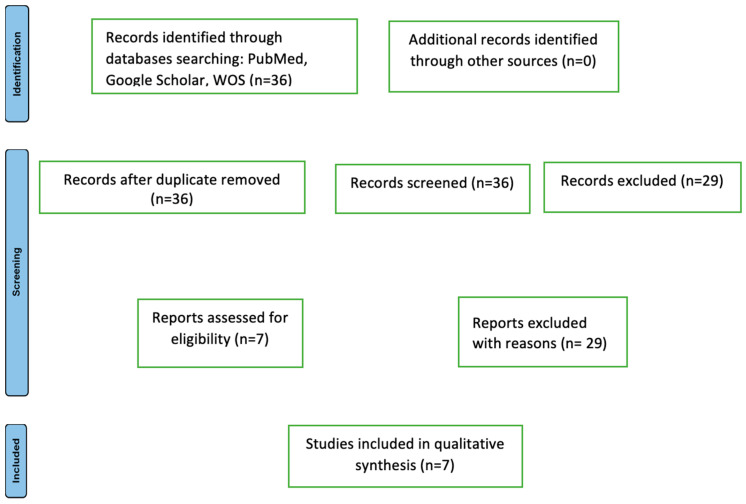
The flowchart of articles selected for the review (n = number).

During the initial research phase, 36 potentially relevant articles were identified for the current study. One additional article in a language other than English was identified but not included in the study. Articles focusing on pediatric cases were excluded. Among the 37 articles, 29 did not contain cases focusing on compartment syndrome of the thoracic limb in cutaneous anthrax (Figure 14). 

The Preferred Reporting Items for Systematic Reviews and Meta-Analysis (PRISMA) 2020 flow diagram for new systematic reviews, which includes the searches of databases and registers only, is shown in Figure 14.

A total of seven publications (2008–2023) met the inclusion criteria for the current study. These seven reports included a total of nine cases in which the cutaneous form of anthrax, with typical lesion localization on a thoracic limb, was complicated by compartment syndrome (Table 4).

The included reports registered the presence of compartment syndrome in nine cases. Among these, eight patients were male and one female (11.11%). The average age of the patients was 47.5 years. In six cases (66.6%), contamination occurred through contact with sick or dead animals; in one case (11.11%), an insect sting was reported; and in 2 cases (22.22%), drug users reported intravenous administration (contaminated heroin). The clinical appearance of the lesions in all the cases coincided with the description in the case definition: painless, pruritic papules evolving into vesicles with central necrotic zones, erythema, and edema. Compartment syndrome in all the cases exhibited well-known clinical signs: ischemic pain, cold skin, distal paresthesia, delayed capillary refill, and absence of radial and ulnar artery pulses. 

The onset of cutaneous lesions from the time of contact with the etiological factor ranged from 2 to 10 days, with one case not documented in the literature report. In four cases, cutaneous lesions were present on the right thoracic limb, in four cases on the left thoracic limb, and in one case, this fact was not mentioned. In three cases (33.33%), BA presence was not confirmed in wound secretions either by Gram staining or by molecular techniques, possibly due to antibiotic treatment initiation. Anthrax cases meeting the epidemiological context, clinical symptomatology, and specific cutaneous lesions (pathognomonic criteria) were reported.

In all the cases of cutaneous anthrax complicated by compartment syndrome of the thoracic limb, an emergency surgical intervention consisting of a fasciotomy was performed. Reconstruction methods were used in two out of the nine cases, performed at varying intervals from the fasciotomy, involving split-thickness skin grafts. Emergency antibiotic treatment was initiated in all the cases, with penicillin being used in four cases.

## 4. Discussion

Cutaneous anthrax, the most common form of anthrax in humans before immunization, previously had a mortality rate ranging from 10 to 40% [19]. Currently, the presence of modern therapies reduces mortality from cutaneous anthrax to less than 1%, with compartment syndrome and toxic shock being frequent causes [20]. Anthrax can occur anywhere on the globe, with its spores being highly resistant and surviving for years in the soil, contaminating grazing livestock [21]. The literature indicates that between 1955 and 2014, 210,111 cases of anthrax were recorded. This number includes both confirmed and probable cases, with a recorded mortality of 3.6% [22].

Endemic areas for anthrax include Sub-Saharan Africa, China, Eastern Europe, Asia, and North America. Anthrax is considered endemic in India, Bangladesh, and Eastern Turkey [5]. Unfortunately, due to drug consumption, a relatively large number of anthrax cases have begun to appear in Western Europe, most of which were reported among intravenous drug users [6,23]. The first case of death from anthrax in a drug user was registered in 2000 in Norway, where the illness was caused by the use of contaminated heroin [6,24]. Following this report, the notion of “injectional anthrax” was introduced into the literature [6]. Cases of injectional anthrax increased with further reports in Scotland (47 cases with 13 deaths) in 2009 [24]; in 2010, Booth et al. reported 31 cases of anthrax in drug users, one of which was reported to have compartment syndrome [18]. In Romania, a report from 2021 by Dumitrescu et al. is recorded, including three cases, one of which was complicated with compartment syndrome. In this particular report, it was the fact that two of the three cases (a woman and a man/husband and wife) declared a history of insect stings, although the third case was a farm worker of the two who also declared contact with a dead goat. This study includes the only case of compartment syndrome recorded in a female patient [10]. In the group reported in the current study, all the patients were male.

Apart from cutaneous anthrax, there are two other forms of anthrax: gastrointestinal, with a mortality between 25 and 75%, and respiratory (pulmonary), with a mortality of 92%. In the event of complications such as meningoencephalitis, the mortality rate is 100% [15]. In the case of cutaneous anthrax, the anatomical regions most exposed to infection are the limbs, head (face), and neck [15].

In order to facilitate diagnosis, definitions were developed for a suspected case of anthrax and a confirmed case of anthrax [25] in humans. A suspected case of anthrax is a person who came into direct contact with sick or dead animals or their products (meat, fur, skin, etc.) or ingested their meat, or a person who inhaled dust contaminated with BA spores or injected himself with drugs contaminated with anthrax. All of these cases show the appearance of macules, itchy, painless papules, located on the chest, face, and/or neck, lesions that over time show blisters, edema, and/or erythema, as well as ulcers or eschars whose dimensions grow in a relatively short time [26]. A confirmed case of anthrax is a case in which BA is isolated in cultures or during PCR testing. According to these recommendations and the current study, there were four suspected and two confirmed cases, the source of the infection was dead animals, whose contamination with anthrax was confirmed before the confirmation in the two patients in whom the disease was complicated with compartment syndrome.

The rate of positive cultures in patients suspected of anthrax was 60% as a result of the early institution of antibiotic treatment [15]. In the case of the four patients reported in the current study, the absence of growth can be attributed to early antibiotic treatment, indicated only as a result of the presence and appearance of the skin lesions as well as the history of the disease, as stated by the patients. Parlak, for his part, reported the group of three patients with compartment syndrome, but only in one case confirmed the presence of BA in cultures [15].

In cutaneous anthrax, incisions, in the early stages of the disease, are not recommended due to the increased potential for septicemia or septic shock [10]. In all cases diagnosed with compartment syndrome, including in the current study, as in the case of compartment syndromes of other etiologies, the surgical indication is an absolute and urgent one. In the case of anthrax, this complication can endanger the patient’s life and be the cause of death. In all cases, fasciotomies are indicated [16]. In both cases presented, the surgical intervention was performed under general anesthesia with orotracheal intubation (OTI). In none of the cases was the problem of loco-regional anesthesia due to the erythema and edema extended to the armpit (especially in the first case), and at the same time, the general anesthesia ensured the possibility of faster establishment of rebalancing measures in case of complications appearing during surgery.

In all the cases, both confirmed and suspected, emergency antibiotic treatment was instituted. In the two cases where surgical intervention was performed for compartment syndrome, Penicillin (6 million IU/day) and Ciprofloxacin (400 mg three times daily) were administered for a period of 10 days, after which, at the recommendation of the infectious disease specialist, the Penicillin was replaced with amoxicillin 1.2 g three times daily for an additional 7 days. As in the case of recommendations from the literature, reconstruction was performed in the described cases within 7–10 days following fasciotomy [15]. The results were favorable, with no deaths recorded. Postoperative functional sequelae did not result in functional impairment of any anatomical segment. However, aesthetic sequelae were observed. Even spontaneous healing of cutaneous anthrax lesions, regardless of size, resulted in visible scars. In cases of compartment syndrome, patient hospitalization is prolonged with relatively slow healing due to significant exudation, edema persisting for 4–5 days despite general anti-edema treatment, and the recommendation for reconstructive techniques to be performed 7–10 days after the initial surgical intervention until the compartment syndrome was resolved. Patients with cutaneous anthrax and compartment syndrome should be monitored, even after discharge, with continued antibiotic treatment for 7 days.

The emergence of a “new” form of anthrax, transmitted through injection, should not be overlooked [27,28]. In cases of the appearance of cutaneous lesions, such as pruritic papules, with edema, erythema, and central necrosis, in known patients or those who report intravenous drug use, the possibility of cutaneous anthrax should be considered. An important, defining role in the diagnosis of cutaneous anthrax is the patient’s medical history, from which we can ascertain the patient’s belonging to risk groups (veterinarians, butchers, animal husbandry workers, etc.), or contact with sick or dead animals of unknown causes, or their products [15].

## 5. Conclusions

The disease and its signs determined as cutaneous anthrax must be known by specialists in all medical specialties, both in endemic areas and worldwide. Early recognition of the disease, its characteristic cutaneous lesions, and compartment syndrome require rapid initiation of antibiotic treatment along with urgent surgical treatment for fasciotomy, which is the lifesaving solution for these cases. Because the World Health Organization (WHO) mentions *Bacillus anthracis* as one of the most important agents that can be used in bioterrorism, even in the case of the disease occurring naturally, such cases must be reported. The presence and detection of cutaneous anthrax are public health issues, individual health concerns, and veterinary medicine issues, and can become national and international security concerns.

## Figures and Tables

**Table 1:** List of search terms entered into the PubMed, Google Scholar, and Web of Science databases for identification of the studies for this systematic review.

	Search Terms
1	Cutaneous anthrax
2	Compartment syndrome
3	1 and 2
4	English
5	01.01.2008 to 31.12.2023

**Table 2:** Evolution of biological constants—case report 1.

	Day 1	Day 4	Day 17
WBC	19.73	19.81	5.85
CRP	1.24	-	0.39
% neutrophils	18.15	16.02	2.9

WBC—white blood cell, CRP—C-reactive protein.

**Table 3:** Epidemiological, clinical, and treatment aspects of cases 3-6, directed to the hospital for infectious diseases in the absence of local complications such as compartment syndrome.

	Age	Sex	Occupation	History	Single/Multiple Cutaneous Lesions + Location Site	Clinical Diagnosis	Paraclinical Diagnosis	Microbiology	Treatment	Evolution
Case 3	38	M	Animal farm worker	7 days	Multiple lesions on the left forearm	Characteristics of skin lesions and epidemiological context, fever, no CS	Increased CRP, leukocytosis	The presence of BA has not been confirmed	Ciprofloxacin, Cefort, no surgery	Favorable evolution, self-limitation of skin lesions
Case 4	45	M	Animal farm worker	7 days	Single injury on the right forearm	Characteristics of skin lesions and epidemiological context, fever, no CS	Increased CRP, leukocytosis	The presence of BA has not been confirmed	Ciprofloxacin, Cefort, no surgery	Favorable evolution, self-limitation of skin lesions
Case 5	52	M	Animal farm worker	7 days	Multiple lesions in both thoracic limbs	Characteristics of skin lesions and epidemiological context, fever, no CS	Increased CRP, leukocytosis	The presence of BA has not been confirmed	Ciprofloxacin, Cefort, no surgery	Favorable evolution, self-limitation of skin lesions
Case 6	48	M	Animal farm worker	7days	Multiple lesions in both thoracic limbs	Characteristics of skin lesions and epidemiological context, fever, no CS	Increased CRP, leukocytosis	The presence of BA has not been confirmed	Ciprofloxacin, Cefort, no surgery	Favorable evolution, self-limitation of skin lesions

M = male, CS = compartment syndrome, BA = *bacillus anthracis*.

**Table 4:** Parameters of the included studies in the systematic review that presented compartment syndrome.

	Year	No. of Cases in the Study	Age	Sex	Source	History	Lesion Localization	Lesion Characteristics	Diagnosis	Treatment
1	2013 Parlak et al. [15]	3	50 y	M	Contact with sick bovine animal	10 days	Left arm	Pruritic pustule erythema, edema, crusted hemorrhagic bullae scar, CS	BacillusG+ in the lesions smears	Penicillin G, fasciotomy
			41 y	M	Contact with sick bovine animal	7 days	Left arm	Pruritic pustule-like, erythema, panicula, bullae with necrotic center, edema, CS	No microorganism	Penicillin, fasciotomy
			35 y	M	Works in husbandry	-	Left hand, left forearm	Erythematous lesions, edema, disseminated bullae cyanosed, crusted lesions, CS	No microorganism	Penicillin G, no surgery
2	2019 Zhao et al. [13]	1	45 y	M	Contact with a dead cow	-	Right upper limb	Cutaneous rash, increased in size and ulcerated, CS	BA nucleic acid +	Meropemen, Clindamycin, Levofloxacin, fasciotomy
3	2011 Parcell et al. [6]	1	28 y	F	Intravenous drug user	2 days	Right arm, right shoulder	Erythema, pain, swelling, CS	Confirmed on histopathology Gram + PCR + for BA	Benzylpenicil, ciprofloxacin, Metronidazole, fasciotomy
4	2017 Kilinc et al. [16]	1	43 y	M	Contact with a dead animal	10 days	Left arm	Swelling, pain, redness hemorrhagic bullous lesions CS	Wound culture result -	Penicillin, Ciprofloxacin, fasciotomy, skin graft
5	2021 Dumitrescu et al. [10]	3	44 y	M	Insect bite	10 days	Right upper limb	Edema, malignant pustule, no CS	Serology for BA + after 12 days	
			44 y	F	Insect bite	-	Right upper limb	Massive edema with blisters, CS	BA + on Gram-stained smear and in blood agar culture Serology for BA + after 12 days	Meropenem, ciprofloxacin, vancomycin, fasciotomy and reconstruction
			41 y	M	Contact with a dead goat	5 days	Both forearms	Malignant pustule, no CS	Serology for BA negative at admission and after 12 days	Ciprofloxacin, Penicillin, vancomycin
6	2010 Knox et al. [17]	3	44 y	M	Intravenous drug user	2 days	Right upper limb	Erythema, Swelling, CS	PCR +	Benzypenicilin, Flucloxacilin, clindamycin, fasciotomy
			36 y	M	Intravenous drug user	3 days	Left forearm	Swelling No CS	Tissue samples tested positive by polymerase chain reaction for *Bacillus anthracis* but Gram stain revealed only scanty pus cells and culture was negative.	Ciprofloxacin, clindamicin, benzylpenicillin, metroniadazole, pre-emptive fasciotomy
			32 y	F	Intravenous drug user	-	Both lower limbs, both hands	Swelling pain, cellulite, abscesses with a central necrotic plug, no CS	PCR +	Antibiotics, debridation, necrectomy Split skin graft,
7	2010 Booth et al. [18]	31 cases 1 case with CS	-	-	Heroin user	-	Upper limb	Edema, necrosis	Gram stain, culture, and PCR +	antibiotics, fasciotomy

y = year, M = male, F = female, CS = compartment syndrome, BA = bacillus anthracis, no. = number.

## Data Availability

The raw data supporting the conclusions in this article will be made available by the authors upon request.
